# COPD: adherence to therapy

**DOI:** 10.1186/2049-6958-9-60

**Published:** 2014-11-22

**Authors:** Alessandro Sanduzzi, Piero Balbo, Piero Candoli, Giousuè A Catapano, Paola Contini, Alessio Mattei, Giovanni Puglisi, Luigi Santoiemma, Anna A Stanziola

**Affiliations:** Department of Respiratory Diseases, Monaldi Hospital, University Federico II, Naples, Italy; Pneumology Thoracic Unit, Ospedale Maggiore della Carità, Novara, Italy; Pulmonary and Endoscopic Thoracic Unit, AUSL, Ravenna, Italy; Clinical Researcher, Respiratory Diseases, Institute of Clinical Physiology, G. Monasterio Tuscany Foundation/National Research Council, Pisa, Italy; Department of Respiratory Diseases, Bellaria Hospital, Bologna, Italy; Department of Pulmonary Diseases, Città della Salute e della Scienza Molinette, Torino, Italy; Department of Pulmonary Diseases, S. Camillo - Forlanini Hospital, Rome, Italy; Pharmacology Specialist, ASL, Bari, Italy

**Keywords:** Adherence, COPD, Inhaler, LABA, LAMA, Questionnaire

## Abstract

Adherence to medical therapies is a growing issue, so much so that the World Health Organization defined it as “a new pharmacological problem”. The main factors affecting compliance are: frequency of administration, rapid onset of action, role of device. The most severe consequence of non-adherence is the increased risk of poor clinical outcome, associated with worsening of the quality of life and increase in health-care expenditure. It appears crucial to identify those COPD patients who are “poorly or not at all compliant with their treatment”. In order to evaluate adherence to the medical therapy, several methods were proposed, the most effective of which turned out to be self-reports, i.e. simple, brief questionnaires (e.g. Morisky test). To increase the likelihood of quickly identifying non-compliant patients, it may be useful to administer a simple questionnaire to naïve subjects (for example, in the waiting room before an examination) including six specific items allowing to identify the patient’s key characteristics. Depending on the answers, patients who do not comply with their pharmacological treatment may be classified as belonging to 6 phenotypes. For patients who are already under treatment it might be useful to administer another short questionnaire during follow up examination. Once the risk of non-compliance is identified, four possible types of measures can be taken: prescription-related, educational, behavioral and complex combined measures (combination of two or more actions).

Therefore, while it is clear that adherence in COPD is a critical issue, it is also obvious that raising awareness on the disease and improving cooperation among specialists, general practitioners, health-care professionals, and patients is the starting point at which this evolution should immediately begin. Each medication is able to foster good compliance with the therapy, and consequently to maximize the efficacy, by virtue of its specific inhaler and its own active ingredient.

**Electronic supplementary material:**

The online version of this article (doi:10.1186/2049-6958-9-60) contains supplementary material, which is available to authorized users.

## Introduction

“Drugs don’t work in patients who don’t take them”. This quote by C. Everett Koop is not so obvious as it might seem. As a matter of fact, in order for a drug to have a therapeutic effect, not only the active ingredient should be effective and the carrier delivering it to the body be optimal, but, most importantly, the patient must adhere to the therapy [1].

The term “compliance “has been used to assess a treatment regimen and the following *passive* behavior from the patient respect to specific prescription like an acquiescent recipient of expert medical prescription.

The accurate definition instead of “adherence” considers the approval and “active” leading part of the patient in his own health management. The latter term of adherence more accurately reflects a patient’ s active role in consenting and following prescribed treatments [2].

Most recently the term “concordance” has been used to describe the “therapeutic alliance” that exists between patients and medical healthcare professionals. In sum, the compliance is a patient’s behavior versus therapeutic prescription; whereas adherence: the patient’s behavior after approval prescription and concordance is the relationship doctor-patient versus agreed decision. Another word has been recently added to the two mentioned above, namely “agreement”, which indicates a medical treatment prescription model based on negotiation and agreement by and between the physician and the patient, reflecting and valuing the viewpoint of the latter.

## Review

In a recent survey carried out with pulmunologists, the definition of adherence in Respiratory Medicine that has received the highest consensus has been the following: “A condition that can only be fulfilled when the patient accepts the presence of the disease and the issues related to the recommended therapy” [3].

Intentional non adherence is an active decision taken by patients who fail to fill prescribed therapy. Approximately 15% of patients do not fill a new prescription and generally discontinue therapy after about six months.

Unintentional non-adherence, instead, could be considered a passive process; the patients fail to adhere to prescribing instructions for many reasons out of their control (old age, social conditions etc.,). Prevalence estimates of unintentional non-adherence vary considerably, but this condition is more frequent with a range from 20% to over 50%. Although it has been documented that patients exhibit both intentional and unintentional non-adherence, no previous study has explored the interrelationship between unintentional and intentional non-adherence in relation to patients’ medication beliefs [4].

Regardless of the language used, it is unquestionable that the full benefits of a therapy can only be reaped if the patient complies as closely as possible with the physician’s prescription.

## Methodological remarks

This paper is the result of the work carried out by a team of 10 experts focusing on Chronic Obstructive Pulmonary Disease (COPD) who developed evaluations and suggestions on inhaled therapies. These experts held several meetings in 2013 in order to discuss the critical issues relating to the administration of inhaled drugs and to the use of the required inhalers.

Throughout the process, the literature available on this topic was examined (over 50 peer-reviewed articles and reviews) [1–38] before the resulting document was discussed and approved in its final version. Then, two questionnaires were developed in order to assess the risk of poor compliance by COPD patients, both naïve patients and patients who were already under inhaled therapy.

The distinctive feature of this work was the observation of real-life scenarios aimed at deriving indications for the application of patient-centered medicine.

The Team hopes that such observations will generate stimuli to develop experiences aimed at validating the above-mentioned questionnaires and then implementing them as part of good clinical practice.

## Adherence: an increasingly serious issue

Adherence to medical therapies is a growing issue, so much so that the World Health Organization (WHO), through Mr. Clancy (Director of the Agency for Health Research and Quality) defined it as “a new pharmacological problem”.

This statement is particularly true if one considers the extent to which non-compliance with a medical therapy may affect the efficacy of a drug also in terms of survival. Some studies even showed that a patient who regularly takes a placebo is more likely to survive than a patient showing poor compliance with a pharmacological therapy [5]. According to the WHO, “maximizing the effectiveness of investments aimed at increasing compliance may have a far better impact on people’s health than any other progress in the therapeutic arena”.

A possible explanation of this unexpected phenomenon could be found in the identification of a “compliant phenotype,” i.e. a person who is more aware of the available health indicators, who takes better care of himself/herself, and follows more closely the therapeutic recommendations given to him/her [6].

**Figure 1:**
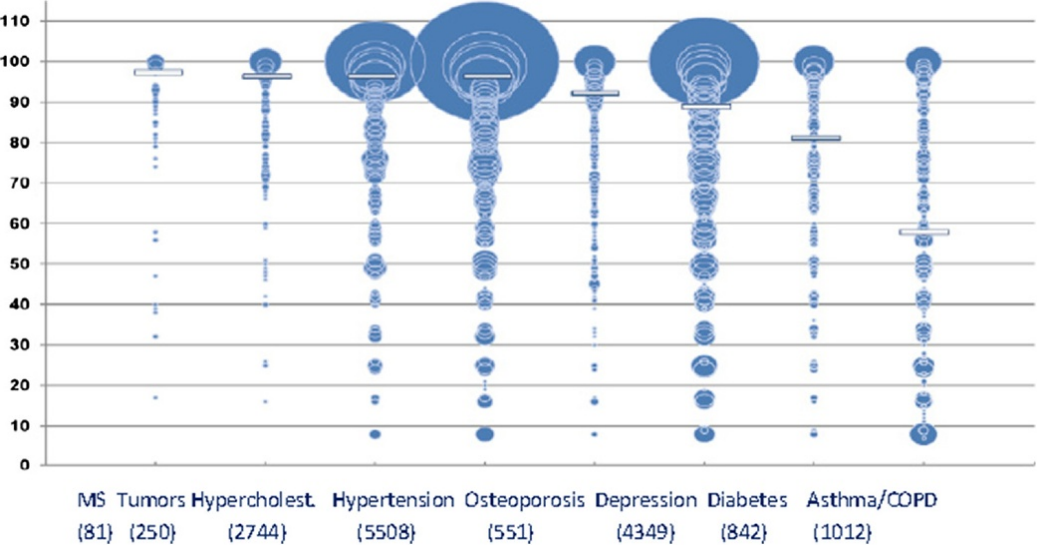
**Distribution of medication possession ratios (MPR) for eight medical conditions among 15,334 patients (the areas of the circles are proportional to the sample sizes.** Median MPR is represented by horizontal bars). Mod. from [9].

Shifting the focus onto COPD, what can be inferred is that, despite the severity of this medical condition, the level of compliance is very low, lower than the compliance rate recorded for other diseases (Figure 1) [1].

In real-life conditions, the percentage of compliant patients is much lower (10-40%) than the percentages reported in the literature (40-60%) [7, 8], and those recorded in clinical trials (70-90%) [9, 10].

Data from a recent Educational Program to stop smoking by the Italian Society of Respiratory Medicine show that the major difficulties are related to the requirement to stop smoking. Unless the patient understands that the first thing to do is to quit smoking, he/she will not understand the importance of strictly following the prescribed medication dosage. Based on the estimates published in a recent DOXA survey, ordered by the three scientific societies SIMER-SIAIC-AAITO, Italy shows some worrying figures. Out of a roughly estimated seven million patients suffering from COPD and asthma (prevalence of 6.2% for asthma and of 5.3% for COPD), 1,000,000 never used spray inhalers, 1,300,000 stopped using them, and 2,700,000 use them “intermittently”, namely as an emergency therapy. On top of this, considering that approximately 300,000 newly diagnosed patients add up to the total amount every year, with an estimated mortality rate of approximately 40,000 patients per year, one can fully understand the extent of the problem [11].

## Causes of non-compliance

Poor compliance with the medical therapy is characterized by a predisposition to unhealthy behaviors and lifestyles and is associated with several factors, which can be correlated with the prescribed medications (number of medications to be taken, difficulties in handling the device, complex regimes, side effects, cost of medications…) or with other causes (old age, physical and cognitive impairment, instructions for use not properly explained or understood, poor trust in the therapies and/or in the physician, weak will, fears or unexpressed problems, underestimation of the severity of the disease, lack of symptoms) [12].

The most common type of non-adherence in COPD patients is underuse and improper use in the most frequent type of non-adherence in patients older than 65 years of age with polypharmacy. The older patients have multiple comorbidities (diabetes, chronic ischemic heart disease, depression, systemic hypertension) and they have to attend a lot of therapeutic prescriptions. Forgetfulness probably is the most common unintentional behavior to skip doses in order to make the medication last longer. The attitude towards inhaled medications in the elderly patients is influenced by a great diversity of factors. The reasons for non-adherence in the older-aged include items related to the medicine (e.g. cost, number of medicines, adverse effects) and those related to the person (e.g. depression) [13]. However, many patients cannot use inhalers correctly, and over 50% of patients struggle to use a metered-dose inhaler properly. Poor inhaler technique is associated with a reduced control, and worst COPD outcomes. A lack of perceived benefit led to 30% of patients with COPD intentionally discontinuing their therapy [14]. Training patients and caregivers in the correct inhaler preparation and use is an essential component in the process toward achieving reliable and repeatable medication delivery. Instructions should be inhaler-specific, and they should include instruction on how to load or prime the device [15].

The WHO grouped these multiple causes of non-compliance into 5 dimensions, i.e. socio-economic, therapy-related, patient-related, disease-related, related to the health-care system or to the medical team [9].

The main factors preventing good compliance include: the patients’ cultural level, psychological issues, insufficient amount of information given at the time of prescription as to the pursued purpose and therapeutic goals, and, most importantly, comorbidities. Indeed, together with the treatment complexity, comorbidities have a bigger negative impact on compliance rather than adverse events [16]. It should be pointed out that, among comorbidities, a major role is played by depression, which affects 10% to 42% of stable COPD patients. A recent study carried out on almost 75,000 patients shows that depression is strongly correlated to the discontinuation of a therapy and to the decrease in the number of days the medication is actually taken by the patient. Hence, the need to promptly start treating this medical condition, before it seriously affects the efficacy of the treatment [17].

However, the Global Initiative on Obstructive Lung Disease (GOLD) recommends that patients with COPD should be referred for pulmonary rehabilitation once their FEV_1_ falls below normal level. Poor patient participation and adherence are the lowest to referring patients with a forced expiratory volume in one second (FEV_1_) < 80% predicted to pulmonary rehabilitation (5%) [18].

## Factors affecting compliance

**Figure 2:**
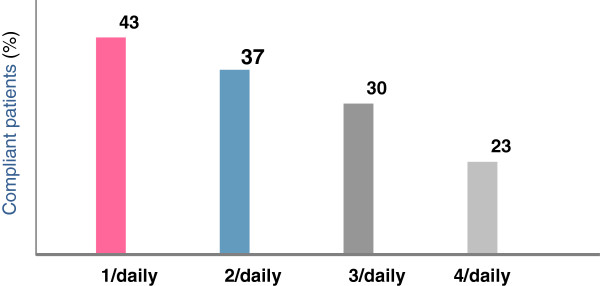
**Percentage of days under treatment as a function of the number of daily administrations in patients with chronic obstructive pulmonary disease.** Mod. from [19].

**Figure 3:**
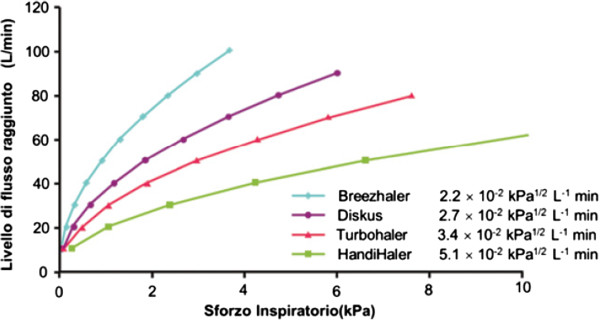
**Resistance to air flow recorded with the main devices.**

1. **Frequency of administration**
  One of the key factors for achieving good compliance is the number of daily administrations. It is actually well known that the higher is the number of administrations, the lower the adherence to the medical therapy. The percentage of compliant COPD patients drops from 43% with a once daily medication to 23% with a therapy requiring 4 administrations per day (Figure 2) [19].
  Therefore, the introduction of once daily medications onto the market is to be viewed as a very positive development. Among the active ingredients that are currently available, indacaterol, glycopyrronium, and tiotropium administered in once daily doses showed an actual 24-hour coverage thanks to their long action, while formetorol, salmetorol, and aclidinium are to be administered twice daily (12 hour coverage only). Looking at the molecules that are currently in the pipeline and will be available in the near future, what can be inferred is that most of them shall come in once daily dosage. The molecules that will become available for use in clinical practice are: Olodaterol, Vilanterol, and Umeclidinium, to be administered as monotherapies, and Indacaterol/Glicopirronium, Vilanterol/Umeclidinium, Olodaterol/Tiotropium, to be administered as combination therapies for the treatment of COPD patients (Table 1).
2. **Rapid onset of action**
  The fast action of a pharmaceutical product can be seen as one of the factors that affect adherence to a medical therapy. It is believed that the perception of the product delivering its action rapidly may lead the patient to continue taking the therapy on a daily basis. Among the products that are currently available, indacaterol, glycopyrronium, and formoterol are fast acting (action starts within 5 minutes), while tiotropium, aclidinium, and salmeterol act in approximately 30 minutes [20].
3. **The role of device**
  Another key factor that is closely related to the patients’ compliance with their medical therapy is the device. If used incorrectly, it remarkably reduces the efficacy of the drug and, consequently, the patient’s compliance. For the treatment of COPD, the most commonly used and preferred inhalers are DPI (dry powder inhalers) [21]. The table below offers an overview of some of the features of inhalers.
  Each COPD medication comes with a dedicated device. Indacaterol and glycopyrronium are administered via Breezhaler (a low-resistance, single-dose DPI), tiotropium via Handihaler (single-dose DPI) or Respimat (soft inhaler), while Aclidinium, Salmetorol/fluticasone, and formetorol/budesonide via multi-dose DPI’s (Genuair, Diskus, Turbohaler, respectively) (Table 2) [22].
  As far as the inherent characteristics of inhalers are concerned, it should always be kept in mind that in the most severe stages of the disease, the inhaler may fail to activate due to an inappropriate peak inspiratory flow, which would prevent an effective dose of drug from reaching the lungs [23]. The flow level achieved depends on the lower resistance that every device makes to the air flow during inhalation (Table 3, Figure 3). A comparison of the inherent resistance of the different devices shows that Breezhaler and Diskus have a lower internal resistance of 2.2 and 2.7 [x10^-2^ kPa^½^ L^-1^min], respectively, while such value is higher for Handihaler, Genuair and Turbohaler, equal respectively to 5.1, 3.5, 3.4 [x10^-2^ kPa^½^ L^-1^min] [24].

**Table 1:** **COPD medications subdivided on the basis of the number of daily administrations**

	LABA	LAMA	LABA/LAMA	LABA/ICS
**Once daily**	**Indacaterol**	**Tiotropium**	*Indacaterol/Glicopirronium*	*Vilanterol/Fluticasone*
	*Olodaterol*	**Glicopirronium**	*Vilanterol/Umeclidinium*	
	*Vilanterol*	Umeclidinium	*Olodaterol/Tiotropium*	
**Twice daily**	**Formaterol**	**Aclidinium**	*Formaterol/Aclidinium*	**Salmeterol/Fluticasone**
	**Salmeterol**			**Formaterol/Budesonide**

In bold, products currently available on the market. In italics, products that will be made available soon.

**Table 2:** **Active ingredients available on the market with their related device and dose**

	Breezhaler^®^	Handihaler^®^	Respimat^®^	Genuair^®^	Diskus^®^	Turbohaler^®^
**Active ingredient**	Indacaterol and Glycopyrronium	Tiotropium bromide	Tiotropium bromide	Aclidinium bromide	Salmeterol/fluticasone	Formoterol/budesonide
**Dose in a capsule**	150μg/300μg INDA	18μg	2,5μg	400μg^6^	50μg SAL	400μg BUD
	50μg GLICO				100/250/500μg FP^7^	12μg FORM^8^
**Delivered dose**	120μg/240μg INDA	10μg^9^	2,5μg	375μg^6^	45μg SAL	320μg BUD
	44μg GLICO				90/225/450μg FP^10^	9μg FORM^8^
**Type of device**	SD-DPI	SD-DPI	Soft-inhaler	MD-DPI	MD-DPI	MD-DPI

^®^, trademark.

**Table 3:** **Inherent characteristics of the devices affecting product delivery**

	Breezhaler^®^	Handihaler^®^	Respimat^®^	Genuair^®^	Diskus^®^	Turbohaler^®^
**Intra-thoracic deposition**	39%^14^	22%^14^	52%^15^	30%^16^	8-14%^17^	25-35%^17^
**FPF**	42.6%	9.8%	66%^18^	36,5%	14-24%^17^	44-46%^17^
**Internal resistance**	2.2x10^-2^kPa_½_ L^-1^min	5.1x10^-2^kPa_½_ L^-1^min	Not applicable	3.5x10^-2^kPa_½_ L^-1^min	2.7x10^-2^ kPa_½_ L^-1^min	3.4 x10^-2^kPa_½_ L^-1^min

FPF, Fine particle fraction; ^®^, trademark.

The different air-flow resistance levels offered by the different devices imply different responses to the inspiratory effort with the same level of flow. It is also important that, also at a low flow, the drug releases a suitable quantity of fine particles in order to guarantee a homogeneous distribution in the small airways. A comparison of the values of such fraction (FPF) shows that Breezhaler, Respimat, and Turbohaler release over 40% of the product in fine particles, while Genuair and Handihaler release 30% and 9,8% respectively, and Diskus 14%-24% (Table 3) [25, 26].

As it has been anticipated above, the device through which the active ingredient is released by inhalation and its technical characteristics play a crucial role and may depend on several different factors, but compliance with the medical treatment is equally important. In this respect, the ability to check - on a gustatory, auditory, and especially visual level - the actual inhalation of the product - for instance through transparent capsules (Breezhaler) or by means of a color change in a window (Genuair) - may be an important factor in increasing the patient’s compliance, and may also generate some sort of additive effect to a specific active ingredient due to the fact that the patient is sure that the daily dose was actually delivered (placebo-like effect) (Table 4) [27].

**Table 4:** **Different modalities used in different devices to check that the product was actually delivered**

	Breezhaler^®^	Handihaler^®^	Respimat^®^	Genuair^®^	Diskus^®^	Turbohaler^®^
**Auditory certainty**	YES	NO	NO	YES	NO	NO
**Gustatory certainty**	YES	YES	NO	NO	NO	NO
**Visual certainty**	YES (capsule empty)	NO	Indirect (Plume)	Indirect (window)	NO	NO

^®^, trademark.

Overall, this data shows that not all devices are the same and that the choice of the device may affect the success rate of the therapy as much as the choice of the active ingredient.

## The consequences of non-adherence

Non-compliance with the prescribed medical therapy has a number of negative consequences (Table 5).

**Table 5:** **Negative consequences of non-adherence**

–	Poor control of symptoms
–	Worsening of the quality of life
–	Increasing number of relapses and more frequent need for health-care services (which account for 35-45% of the disease-related costs)
–	Higher mortality rate (2–3 times higher than in patients showing good compliance – 26.4% vs. 11.3% according to a sub-analysis made in the TORCH study [28]
–	Increase in health-care expenditure.

Of course, the most severe factor of such consequences is the increased risk of a poor clinical outcome, although the economic impact also plays an important role. In this respect, it should be pointed out that, in the past few years, the growing use of active ingredients belonging to the LABA and LAMA classes led to a significant improvement in survival after hospitalization due to relapses (data from a Spanish study regarding two cohorts of patients examined after 7 years in the 1996–1997 and 2003–2004 two-year periods), and that a mere 5% improvement in PDC (Proportion of Days Covered) may reduce costs incurred into for examinations delivered in hospital (-2.6%), day hospital (-2%), and Emergency Room (-1.8%) settings [29].

Considering that, in Italy, failure to use inhaled medications for chronic respiratory diseases, or wrong use thereof, leads to a 20% increase in the risk of hospitalization and to a 50% increase in health-care expenditure for overall direct/indirect costs, which amount to €14 billion/year, of which €9 billion are for COPD alone (with an estimated *per capita* cost of €2,723), the extent of the problem is easily understood [30].

In light of the facts outlined above, there is a clear need to raise awareness among the local authorities “(Payor)” as to the fact that the choice of an expensive treatment, provided it is effective and well accepted by the patients, is not only aimed at improving personal wellbeing, but also at reducing the overall expenditure.

## Patient identification and compliance evaluation

It appears crucial to identify those COPD patients who are “poorly or not at all compliant with their treatment,” as if they were a separate clinical phenotype, so as to give these patients the correct indications according to their individual characteristics.

One or more of the following characteristics contribute to defining the profile of non-adherent patients: they are very old, never quit smoking, suffer from mild-moderate COPD, take several different medications, live alone, are depressed, have limited economic resources, show cognitive/cultural deficits, have poor trust in physicians and medications, do not realize the gravity of the disease or don’t want to see themselves as such, justify or hide their symptoms, are reluctant to follow long-term therapies, are well documented on their disease and know that they need to take specific medications but do not take any action and criticize any kind of pharmacological option (evolved inactivity), and sometimes may not understand the Italian language [31].

In order to evaluate adherence to the medical therapy, several methods were proposed, the most effective of which turned out to be self-reports, i.e. simple, brief questionnaires (e.g. Morisky test [32]) that provide a clear overview of the respondents’ degree of compliance with their medical therapy.

To increase the likelihood of quickly identifying non-compliant patients, it may be useful to administer (for example, in the waiting room before an examination) a simple questionnaire including some specific items allowing to identify the patient’s key characteristics [33].

Depending on the answers, patients who do not comply with their pharmacological treatment may be classified as belonging to one of the following 6 phenotypes: 1) patients who consider the treatment too complex, 2) patients with wrong beliefs, 3) patients who are not aware of the importance to follow their prescriptions, 4) patients who doubt the efficacy of their medications, 5) patients who are poorly attentive, and 6) patients who believe that the efforts required to comply with the therapy overweigh the benefits of the therapy itself. Later on, other evaluations follow up to this initial assessment during treatment so as to investigate the reasons underlying any failure to comply with the therapy: wrong interpretation, forgetfulness, skepticism, unconscious reasons (denial of the problem, the drug reminds the patients of their disease), administration route, adverse events, fear of addiction, belief that the disease was finally cured, costs.

At this point, two homogeneous areas can be identified, each including indicators that can be correlated with the risk of poor adherence. The first area is geared towards assessing personal/family-related risk situations and the relationship with medications in general, while the second one is aimed at defining the risks of poor adherence which are directly linked to the relationship that COPD patients already under treatment have with inhaled medications.

### Proposed psycho-socio-economic questionnaire to be administered to naive patients for the identification of their phenotype

In order to find out whether a patient will be compliant with the physician’s prescription, a simple, quick questionnaire with only 6 questions can be administered to naive patients in the waiting room before they are seen by a doctor, which might be even more effective if it were shared with Primary Care Medical Centers since it is not limited to the category of inhaled drugs [10]. The rationale behind the selection of such 6 questions is the result of the following considerations: several studies showed that patients’ compliance is inversely proportional to the number of administrations (Question 1), living with a family member could make it more likely for the patients to remember taking their medications (Question 2), worries, physical disorders and poor compliance with the medical examination schedule have a negative impact on adherence (Questions 3, 4, and 6), the patients’ opinion on inhaled medications is reflected on their consistent use (Question 5) [34]. See Additional file 1.

### Questionnaires for patients who are already under treatment aimed at confirming their adherence status and phenotype

The proposal of this questionnaire is to asses of non-adherence in COPD patients with inhaled medication.

Here again, the indicators are gathered in a short questionnaire designed to be administered to patients who are already under treatment, during their follow up examinations. Based on few, simple questions, this questionnaire may help pulmonologists to identify compliant, poorly compliant, or non-compliant patients, and consequently to take the necessary measures.

The proposed reference questionnaire is again the Morisky scale [32], in a modified version with 3 specific questions on inhaled therapies. See Additional file 2.

#### Suggestions for possible solutions

It should not be underestimated that compliance with therapeutic choices is an indicator of quality of care, and some organizations are already planning to use this aspect through specific indicators (e.g. Centers for Medicare and Medicaid Services Five-Star Quality Rating System) because of its high impact on the quality of life and on health-care policies. The strategies for increasing adherence that were most frequently quoted in a survey conducted among pulmonologists were: to provide clear, simple information on therapy schemes, and to identify markers of non-compliance and defined clusters [35].

Once the risk of non-compliance is identified, the possible corrective actions might be:

- To engage patients in the therapeutic strategy and provide them with information on how to manage difficulties
- To facilitate continuity in the delivery of health-care services by the organization or specialists
- To treat comorbidities, especially depression and anxiety
- To offer near-term follow up examinations to patients at high risk of non-compliance
- To increase interactions between specialists and general practitioners
- To disseminate technical data sheets on inhaled medications in the most commonly spoken languages.

Another factor that should be taken into due consideration, since it turned out to have a significant impact on adherence to the prescribed therapy, is the selection of the device, whose characteristics should be as close as possible to those of an “ideal inhaler”:

- Dose reproducibility
- Operation under low flow peaks
- Lowest resistance to inhalation
- High release of fine particles
- Ability to check that the product was actually delivered.

In essence, four possible types of measures can be taken, namely: prescription-related (simplification of administration and dosage of the medications), educational, behavioral, and complex combined measures (combination of two or more actions).

However, it is unquestionable that compliance with a therapeutic regimen will improve if the patients are able to perceive the severity of their disease and the benefits of their treatment, if they become aware of being at risk of exacerbations/complications, if they establish a good relationship with their physician, and feel able to do what the therapeutic regimen requires so as to develop a good sense of self-efficacy and of *locus* of control.

Moreover, an important role in improving compliance could be played by nurses, who can be put in charge of conducting interviews with the patients before and during treatment in order to motivate them - especially if they are anxious and/or depressed - to adhere to their prescriptions [36].

## Conclusions

In an age of evidence-based medicine, all efforts aimed at reaching a higher level of quality in health care have always been directed towards implementing educational programs targeted to physicians so as to provide correct pharmacological prescriptions with unquestionable, proven efficacy, as demonstrated in large clinical trials regarding outcome, quality of life, and prognostic indicators. On the other hand, over the years, far less emphasis has been put onto the actual compliance with the prescribed medications, which is often considered to be exclusively under the patient’s responsibility.

While it is clear that adherence in COPD is a critical issue, and improving it will certainly take time and require major efforts, it is also obvious that raising awareness of the disease and improving cooperation among specialists, general practitioners, health-care professionals, and patients is the starting point at which this evolution should immediately begin. Reaching the goal of therapy adherence and success also depends on the selection of the device. Each medication with its associated inhaler and its own active ingredient has different characteristics in terms of internal resistance, intra-thoracic deposition, mechanism to check that the product was actually delivered, time and duration of action, which may indeed foster good compliance with the therapy and consequently maximize its efficacy [37].

This paper suggests the dissemination of two questionnaires among health-care professionals so that they can check their usefulness in real-life scenarios, in addition, it makes them available also to the scientific community with a view to conduct further research projects aimed at validating their actual efficacy.

## Electronic supplementary material

Additional file 1:**Questionnaire to estimate the compliance of a naive patient with an inhaled therapy.**(DOC 26 KB)

Additional file 2:**Questionnaires for patients who are already under treatment aimed at confirming their adherence status and phenotype.**(DOC 36 KB)

## References

[1] Raule G (2011). L’aderenza in Medicina Respiratoria. Programma Educazionale della Società Italiana di Medicina Respiratoria. Edizione 2011 del Caleidoscopio Pneumologico.

[2] Osterberg L, Blaschke T (2005). Adherence to medication. N Engl J Med.

[3] Braido F, Baiardini I, Sumberesi M, Blasi F, Canonica GW (2013). Obstructive lung diseases and inhaler treatment: results from a national public pragmatic survey. Respir Res.

[4] Lareau SC, Yawn BP (2010). Improving adherence with inhaler therapy in COPD. Int J Chron Obstruct Pulmon Dis.

[5] World Health Organization (2008). Adherence to long-term therapies: policy for action. Meeting report 4–5 June 2001.

[6] Cartabellotta A (2013). La non-compliance alla terapia farmacologica: strategie diagnostico-terapeutiche. Evid Publ GIMBE Found.

[7] Restrepo RD, Alvarez MT, Wittnebel LD, Sorenson H, Wettstein R, Vines DL, Sikkema-Ortiz J, Gardner DD, Wilkins RL (2008). Medication adherence issues in patients treated for COPD. Int J Chron Obstruct Pulmon Dis.

[8] Charles MS, Blanchette CM, Silver H, Lavallee D, Dalal AA, Mapel D (2010). Adherence to controller therapy for chronic obstructive pulmonary disease: a review. Curr Med Res Opin.

[9] Rolnick SJ, Pawloski PA, Hedblom BD, Asche SE, Bruzek RJ (2013). Patient characteristics associated with medication adherence. Clin Med Res.

[10] Rotter JB (1979). Social Learning and Clinical Psychology.

[11] Dal Negro RW, Micheletto C, Tosatto R, Dionisi M, Turco P, Donner CF (2007). Costs of asthma in Italy: results of the SIRIO (Social Impact of Respiratory Integrated Outcomes) study. Respir Med.

[12] Ostrowski M (2007). Report takes aim at America’s other drug problem: poor adherence. J Fam Pract.

[13] Unni EJ, Farris KB (2011). Unintentional non-adherence and belief in medicines in older adults. Patient Educ Couns.

[14] Giraud V, Roche N (2002). Misuse of corticosteroid metered-dose inhaler is associated with decreased asthma stability. Eur Resp J.

[15] Bandura A (1977). Principles of Behavior Modification.

[16] Melani A, Bonavia M, Cilenti V, Cinti C, Lodi M, Martucci P, Serra M, Scichilone N, Sestini P, Aliani M, Neri M, Gruppo Educazionale Associazione Italiana Pneumologi Ospedalieri (2011). Inhaler mishandling remains common in real life and is associated with reduced disease control. Respir Med.

[17] Qian J, Simoni-Wastila L, Rattinger GB, Zuckerman IH, Lehmann S, Wei YJ, Stuart B (2014). Association between depression and maintenance medication adherence among Medicare beneficiaries with chronic obstructive pulmonary disease. Int J Geriatr Psychiatry.

[18] Sohanpal R, Hooper R, Hames R, Priebe S, Taylor S (2012). Reporting participation rates in studies of non-pharmacological interventions for patients with chronic obstructive pulmonary disease: a systematic review. Syst Rev.

[19] Toy EL, Beaulieu NU, McHale JM, Welland TR, Plauschinat CA, Swensen A, Duh MS (2011). Treatment of COPD: relationships between daily dosing frequency, adherence, resource use, and costs. Respir Med.

[20] Colthorpe P, Voshaar T, Kieckbusch T, Cuoghi E, Jauernig J (2013). Delivery characteristics of a low-resistance dry-powder inhaler used to deliver the long-acting muscarinic antagonist glycopyrronium. J Drug Assess.

[21] Anderson P (2006). Use of Respimat Soft Mist Inhaler in COPD patients. Int J Chron Obstruct Pulmon Dis.

[22] Atkins PJ (2005). Dry powder inhalers: an overview. Respir Care.

[23] Hill LS, Slater AL (1998). A comparison of the performance of two modern multidose dry powder asthma inhalers. Respir Med.

[24] Borgstroem L, Asking L, Thorsson L (2005). Idealhalers or realhalers? A comparison of Diskus and Turbuhaler. Int J Clin Pract.

[25] Magnussen H, Watz H, Zimmermann I, Macht S, Greguletz R, Falques M, Jarreta D, Garcia GE (2009). Peak inspiratory flow through the Genuair inhaler in patients with moderate or severe COPD. Respir Med.

[26] Newman SP, Sutton DJ, Segarra R, Lamarca R, de Miquel G (2009). Lung deposition of aclidinium bromide from Genuair, a multidose dry powder inhaler. Respiration.

[27] Pavkov R, Mueller S, Fiebich K, Singh D, Stowasser F, Pignatelli G, Walter B, Ziegler D, Dalvi M, Dederichs J, Rietveld I (2010). Characteristics of a capsule based dry powder inhaler for the delivery of indacaterol. Curr Med Res Opin.

[28] Calverley PM, Anderson JA, Celli B, Ferguson GT, Jenkins C, Jones PW, Yates JC, Vestbo J, TORCH investigators (2007). Salmeterol and fluticasone propionate and survival in chronic obstructive pulmonary disease. N Engl J Med.

[29] Almagro P, Salvado M, Garcia-Vidal C, Rodriguez-Carballeira M, Delgado M, Barreiro B, Heredia JL, Soriano JB (2010). Recent improvement in long-term survival after a COPD hospitalisation. Thorax.

[30] Dal Negro RW, Tognella S, Tosatto R, Dionisi M, Turco P, Donner CF (2008). Costs of chronic obstructive pulmonary disease (COPD) in Italy: the SIRIO study (social impact of respiratory integrated outcomes). Respir Med.

[31] George J, Kong DC, Thoman R, Stewart K (2005). Factors associated with medication non-adherence in patients with COPD. Chest.

[32] Morisky DE, Green LW, Levine DM (1986). Concurrent and predictive validity of a self-reported measure of medication adherence. Med Care.

[33] Barnestein-Fonseca P, Leiva-Fernández J, Vidal-España F (2011). Is it possible to diagnose the therapeutic adherence of patients with COPD in clinical practice? A cohort study. BMC Pulm Med.

[34] Dalby R, Spallek M, Voshaar T (2004). A review of the development of Respimat Soft MistTM Inhaler. Int J Pharm.

[35] Rosenbaum L, Shrank WH (2013). Taking our medicine - improving adherence in the accountability era. N Engl J Med.

[36] Haynes RB, Montague P, Oliver T, McKibbon KA, Brouwers MC, Kanani R (2000). Interventions for helping patients to follow prescriptions for medications. Cochrane Database Syst Rev.

[37] Van der Palen J, Klein JJ, Van Herwaarden CL, Zielhuis GA, Seydel ER (1999). Multiple inhalers confuse asthma patients. Eur Respir J.

[38] Covvey JR, Mullen AB, Ryan M, Steinke DT, Johnston BF, Wood FT, Boyter AC (2014). A comparison of medication adherence/persistence for asthma and chronic obstructive pulmonary disease in the United Kingdom. Int J ClinPract.

