# Supplementary material for: COPD: adherence to therapy
Source: Multidiscip Respir Med. 2014 Nov 22;9(1):60. doi: 10.1186/2049-6958-9-60 (PMC4256899; doi:10.1186/2049-6958-9-60)
Supplement: Supplementary file 1 — Additional file 1:Questionnaire to estimate the compliance of a naive patient with an inhaled therapy.(DOC 26 KB) [file 40248_2014_185_MOESM1_ESM.doc]

**Additional file 1**

**Questionnaire to estimate the compliance of a naive patient with an inhaled therapy**

Here is the questionnaire to estimate the compliance of a naive patient with an inhaled therapy

1) How many different medications do you take every day?

a- None

b- Less than 5

c- Between 6 and 10

d- More than 10

2) Whom do you live with?

a- Wife/husband and child/children

b- Wife/husband in good health

c- Alone

d- With a family member who is in poor health conditions

3) Are you worried?:

a- No

b- About your health conditions but you are willing to do your best to try and improve them

c- About your health conditions and you do not see any effective, final solution

d- About your economic condition, about your family situation (disabled family member, separated or divorced, no children, children with problems, etc.), about your bad health conditions that by now are unlikely to improve, about your economic situation (unemployed, insufficient pension, cannot pay rent/bills)

4) Would you stop taking a medication if it gave you side effects or if it were too complicated to take?

a- No, absolutely not

b- I would first talk about it with my doctor

c- Yes, if the side effects were particularly disturbing

d- I certainly would

5) What do you think about inhaled medications?

a- They are medicines used to treat chronic respiratory diseases

b- They are not very effective, but occasionally they may be useful

c- They do not bring about any improvement

d- I do not like those containing cortisone because they could be dangerous

6) Do you see any pulmonologists for check-up examinations?

a- Yes, I have been seeing the same physician for a long time now

b- Yes, I go to different doctors to get different opinions

c- Yes, I see different doctors depending on who is available when I call the reservation service

d- No, never. I only see a pulmunologist in urgent situations or in hospital

**Score**

Add up the scores of each answer: A = 3 points B = 2 points C = 1 point D = 0 points.

Score ≤ 12 = patient at risk of poor compliance.

Score > 12 = patient with good probability of adherence.
