# Supplementary material for: COPD: adherence to therapy
Source: Multidiscip Respir Med. 2014 Nov 22;9(1):60. doi: 10.1186/2049-6958-9-60 (PMC4256899; doi:10.1186/2049-6958-9-60)
Supplement: Supplementary file 2 — Additional file 2:Questionnaires for patients who are already under treatment aimed at confirming their adherence status and phenotype.(DOC 36 KB) [file 40248_2014_185_MOESM2_ESM.doc]

**Additional file 2**

**Questionnaires for patients who are already under treatment aimed at confirming their adherence status and phenotype**

1) Do you often forget to take your prescription drugs?

a) Yes

b) No

2) Do you take your medications at the prescribed time?

a) Yes

b) No

3) Do you ever happen to discontinue your therapy when you feel better?

a) Yes

b) No

4) Do you ever happen to discontinue your therapy when you feel worse?

a) Yes

b) No

5) Have you ever discontinued/reduced inhaled medications due to their side effects or fear of possible side effects?

a) Yes

b) No

6) Do you believe that inhaled drugs are appropriate for the therapy of your disease?

a) Yes

b) No

7) Do you prefer oral tablets to inhaled medications?

a) Yes

b) No

Every answer is given a 0 or 1 score (as required for the Morisky scale). Yes =1 No =0 .

**Score**

Good compliance 0–1 Intermediate compliance 2–4 Poor compliance 5-7

| Questionnaire to check patients’ adherence to their inhaled therapy | |
| --- | --- |
| 1) How often did you forget to take your inhaled therapy in the past month? | |
| A) Rarely | B) Often |
| 2) Are you more likely to forget taking your medicines in the morning or in the evening? | |
| A) Morning | B) Evening |
| 3) Do you find your therapy dangerous? | |
| A) Yes | B) No |
| 4) Do you prefer inhaled drugs to oral tablets? | |
| A) Yes | B) No |
| 5) Do you think your prescription is appropriate for your disease? | |
| A) Yes | No |
| Score Add up the scores of the individual answers: A = 2 points B = 0 points (Min. 0; Max. 10) Score ≤ 6 = patient with poor compliance. Score > 6 = patient with good compliance. | |
